# Preparation and Improvement of Physicochemical and Functional Properties of Dietary Fiber from Corn Cob Fermented by *Aspergillus niger*

**DOI:** 10.4014/jmb.2308.08010

**Published:** 2023-10-16

**Authors:** Yadi Zhou, Qijie Sun, Chao Teng, Mingchun Zhou, Guangsen Fan, Penghui Qu

**Affiliations:** 1 Key Laboratory of Green Manufacturing and Synthetic Biology of Food Bioactive Substances, China General Chamber of Commerce, Beijing Technology and Business University, No. 11 Fucheng Street, Haidian District, Beijing 100084, P.R. China; 2 School of Food and Health, Beijing Technology and Business University, No. 11 Fucheng Street, Haidian District, Beijing 100084, P.R. China; 3 Beijing Engineering and Technology Research Center of Food Additives, Beijing Technology and Business University, No. 11 Fucheng Street, Haidian District, Beijing 100084, P.R. China

**Keywords:** Corn cob, fermentation, soluble dietary fiber, functional properties, *Aspergillus niger*

## Abstract

Corn cobs were fermented with *Aspergillus niger* to produce soluble dietary fiber (SDF) of high quality and excellent food safety. In this work, the fermentation process was optimized by single-factor test and response surface methodology (RSM). The optimal fermentation conditions were determined to be a material-liquid ratio of 1:30, an inoculum concentration of 11%, a temperature of 32°C, a time of 6 days, and a shaking speed of 200 r/min. Under these conditions, the SDF yield of corn cob increased from 2.34% to 11.92%, and the ratio of soluble dietary fiber to total dietary fiber (SDF/TDF) reached 19.08%, meeting the requirements for high-quality dietary fiber (SDF/TDF of more than 10%). Scanning electron microscopy (SEM) and Fourier-transformed infrared spectroscopy (FT-IR) analysis revealed that the fermentation effectively degraded part of cellulose and hemicellulose, resulting in the formation of a loose and porous structure. After fermentation the water swelling capacity, water-holding capacity, and oil-holding capacity of the corn cob SDF were significantly improved and the adsorption capacity of glucose, cholesterol, and nitrite ions all increased by more than 20%. Moreover, the total phenolic content increased by 20.96%, which correlated with the higher antioxidant activity of SDF. Overall, the fermentation of corn cobs by *A. niger* increased the yield and enhanced the functional properties of dietary fiber (DF) as well.

## Introduction

In recent years, the lack of dietary fiber in peoplés dietary structure has led to the widespread occurrence of various unhealthy diet-induced subhealth diseases. Dietary fiber (DF) is a class of edible carbohydrates that cannot be digested and absorbed by endogenous enzymes in the human small intestine, but can be partially or completely fermented in the large intestine. DF can promote intestinal peristalsis, effectively reduce serum cholesterol levels and glucose absorption, increase minerals absorption, eliminate exogenous harmful substances, and reduce the risk of colon cancer and cardiovascular diseases, etc. [1]. DF is divided into soluble dietary fiber (SDF) and insoluble dietary fiber (IDF), the sum of which is collectively known as total dietary fiber (TDF), which differ in composition and physiological activity. Compared to IDF, SDF has superior physicochemical properties and physiological activity, including solubility, water-holding capacity, oil-holding capacity, adsorption capacity, and antioxidant capacity. Approximately 20%-30% of human dietary fiber intake should come from soluble dietary fiber. Therefore, good quality dietary fiber often consists of high levels of soluble dietary fiber [2]. High-quality DF generally contains more than 10% SDF, which is widely used as an additive in the food industry [3].

Corn is one of the world's major food crops, with a total global production of 1.086 billion tons in 2022 [4]. About two-thirds of Asia's corn acreage is concentrated in China. As a multifunctional crop, corn is not only primarily used as livestock feed in the world but also serves as an industrial and energy crop. Corn cobs are abundant by-product of corn processing, with 18 kg produced for every 100 kg of corn kernels processed. They are abundant, easily accessible, safe, and non-toxic. Corn cobs are rich in nutrients, mainly composed of cellulose, hemicellulose, lignin, and ash, of which cellulose and hemicellulose account for about 70% [5], and are a representative and rich dietary fiber resource. However, current exploitation of corn cob is mainly focused on the production of xylan, xylitol, furfural, etc. [6]. As a significant agricultural waste, corn cobs have great potential and value for being converted them into high-quality dietary fiber.

The preparation of DF from corn cobs mainly adopts chemical methods, enzymatic methods, or methods combined with ultrasound, microwave, and other emerging technologies. The chemical method requires the use of acid, alkali, or a combination of acid and alkali to treat the raw materials, which is not only demanding on the equipment but also pollutes the environment with the waste liquid generated during the operation. Additionally, the physiological activity of the extracted DF will be lost, limiting its industrial application somewhat [7]. In addition, the high cost of enzyme production in the enzymatic method prevents its widespread use in the actual food industry [6].

In contrast, microbial fermentation is considered to be a safe, effective, and low-cost method for the preparation and enhancement of high-quality DF [8]. The principle of microbial fermentation is essentially the same as that of enzyme preparation of DF. The difference is that microbial fermentation saves the separation and purification process of enzyme, reduces the cost of production, and the production, and the simplification of the production makes it easier be promoted to industrial scale production [9]. In addition, it also solves the problem of hydrolysis product treatment in the fermentation process [2]. The microbial fermentation method is simple in operation, inexpensive, and easy to scale up for large-scale production.

Fermentation has the advantage of being environmentally favorable and efficient as a method to prepare and enhance high-quality DF, and the selection of strains is crucial in the preparation of DF by fermentation. The strains commonly used in fermentation include *Lactobacillus bulgaricus* and *Streptococcus thermophilus*, *Bacillus natto*, *Ganoderma lucidum*, *Trichoderma viride*, and *Aspergillus niger*, etc. [3, 9]. Numerous studies have shown that fungal strains have a high capacity to degrade cellulose and hemicellulose, and can also secrete various enzymes, such as amylase and protease. In addition, the fungal polysaccharides produced during the fermentation process are a good source of dietary fiber. *A. niger* is a non-pathogenic filamentous fungus commonly used in food fermentation and has a recognized safety profile [10]. In the fermentation process, it can produce cellulase, hemicellulase, xylanase, pectinase, and other abundant enzyme systems, as well as a variety of metabolites, which are often used in food production due to their health-related properties [11]. By increasing SDF content, short-chain fatty acids produced by fermentation can improve the physicochemical characteristics of DF, such as hydraulic and oil-holding power. To our knowledge, there was still no research on the preparation of high-quality DF from corn cob fermented by *A. niger*.

In our study, an edible fungus *A. niger* was used to prepare high-quality corn cob DF. The fermentation conditions were optimized to improve the yield of corn cob SDF. In addition, the changes in composition, structure, physicochemical, and functional properties of corn cob SDF before and after fermentation were evaluated.

## Materials and Methods

### Materials

Corn cobs, supplied by Shandong Longli Biotechnology Co., Ltd (China), were dried to constant weight, crushed, and passed through 60 mesh sieve. Xylose, glucose, bovine serum protein, and other standard products were purchased from Sigma Co., Ltd., USA. Thermally stable alpha-amylase, papain, Saccharifying enzyme, and Beech xylan were purchased from Aladdin Chemistry Co., Ltd (China). Other chemicals and reagents were analytical grades, and obtained from Shanghai Yuanye Biotechnology Co., Ltd. (China).

### Strains

*A. niger* F0607, *A. niger* F0609, and *A. niger* F06012 were conserved from Beijing Food Nutrition and Human Health Highly Advanced Innovation Center; *A. niger* 3.1858, *A. niger* 3.2103, etc., were purchased from China Microbial Strain Conservation Center.

### Preparation of Corn Cob Dietary Fiber

Ten percent activated seed solution in PDA medium was inoculated into the fermentation medium and incubated for four days at 28°C and 150 r/min. The fermentation broth was filtered by centrifugation and the sediment was freeze-dried and pulverized to obtain corn cob IDF. The supernatant was added with 4 times the volume of 95% ethanol, chilled at 4°C for 12 h, and then centrifuged. The sediment was freeze-dried and pulverized to obtain corn cob SDF. Corn cob TDF was obtained by combining IDF and SDF. The SDF was extracted directly from corn cob by AOAC (2000) [12] method as a control.

### Optimization of the Fermentation Process

The corn cob material-liquid ratio (1:10-1:35 g/mL), inoculum concentration (6-14%, (V/V)), fermentation time (3-8 d), fermentation temperature (25-45°C), shaking speed (50-250 r/min) and initial pH (3.0-8.0) of fermentation were selected in turn. The SDF yield after fermentation was measured and calculated. The fermentation process was optimized using a three-factor three-level Box-Behnken experiment (BBD) based on single-factor experiments. The experimental design was shown in Table 2.

### Fermentation Process Dynamic Monitoring

Carboxymethyl cellulase activity and xylanase activity were determined following Teng *et al*. [6], while amylase activity was determined according to Nazarova *et al*. [13]. Reducing sugar content was measured using the DNS method [9]. The total sugar content was quantified using the phenol-sulfuric acid method, while the soluble protein content was determined by the Komas Brilliant Blue colorimetric assay [14, 15].

### Monosaccharide Composition

The monosaccharides compositions of corn cob DF were determined by Agilent 1260 HPLC system (Agilent Technologies, USA) equipped with Zorbax-Eclipse Plus C18 column (4.6 × 250 mm i.d.,5 μm) and a UV detector according to the method of Wang *et al*. [16] with slight modifications. Briefly, a binary mobile phase consisting of A-20 mM Na-phosphate buffer pH 6.9 and B-acetonitrile, was used in gradient in 82:18 (V/V) isocratic. The signals of 10 μl pre-filtered samples (0.2 μm, regenerated cellulose) were monitored at the column temperature of 30°C and 250 nm at a flow rate of 1.0 ml/min for 40 min. All samples and monosaccharide standards of mannose, galacturonic acid, glucuronic acid, glucose, rhamnose, galactose, xylose and arabinose were subjected to a derivatization procedure as described previously before HPLC-PDA analysis [16].

### Scanning Electron Microscopy (SEM)

Scanning electron microscopy (SEM) SU8010 (Hitachi Limited, Japanese) was used to observe the structural characteristics of fermented corn cob soluble dietary fiber (F-SDF) and directly extracted corn cob soluble dietary fiber (B-SDF). The samples were placed on the conductive gel, put on the carrier table of SEM and covered with a thin layer of gold produced by the sputter coater. The samples were analyzed using SEM at an accelerating potential of 10 kV and under high vacuum conditions, and micrographs were recorded at different magnifications.

### Fourier-Transformed Infrared Spectroscopy (FT-IR)

Fourier-transformed infrared spectroscopy was performed with NICOLET iS50 FT-IR (Thermo Scientific, USA). 1-2 mg samples were fully mixed with potassium bromide and made into particles. After grinding and pressing, the samples were put into the sample tank for infrared analysis with a spectral resolution of 4 cm^-1^. FT-IR spectra were recorded from 400 to 4000 cm^-1^.

### Physicochemical Properties

Water holding capacity (WHC) was estimated following the method of Raghavendra *et al*. [17], Oil holding capacity (OHC) and water swelling capacity (WSC) was determined respectively according to the method reported by Zhang *et al*. [18] and Zhang *et al*. [19].

### Glucose Adsorption Capacity and Cholesterol Adsorption Capacity

The Glucose adsorption capacity (GAC) and Cholesterol adsorption capacity (CAC) were determined by the method of Kabir *et al*. [20] and Zhang *et al*. [21], respectively.

### Nitrite Ion Adsorption Capacity

Determination of nitrite ion adsorption capacity (NAC) was adopted by the method of Zhu *et al*. [22] with minor modifications. The dried SDF samples (0.5 g) were thoroughly mixed with 100 ml of 100 μmol/l NaNO_2_ solution, and the pH was adjusted to 2.0 and 7.0, to simulate the environment of the stomach and intestine, respectively. The mixture was incubated at 37°C for a certain time under continuous stirring. Then, absorbance was measured at 538 nm and quantified according to a standard curve. The NAC was calculated as follows:

$$NAC(\mu g/g)=\frac{(C_{1}-C_{2})\times M}{m\times V}$$ (1)

Where C_1_ and C_2_ are the concentration of NaNO_2_ solution before and after adsorption, μmol/l; M is the relative molecular weight of NaNO_2_, g/mol; m is the weight of DF, g; V is the total volume of processed samples, L.

### DPPH Free Radical Scavenging Capacity

DPPH free radical scavenging capacity was estimated following a slightly modified method of Ma *et al*. [23]. 100 μl DPPH-ethanol (0.5 mM) was mixed with 100 μl SDF solution with different concentrations. The solution was placed in the dark for 30 min and kept shaking. The absorbance of the sample was measured at 517 nm. The Free radical scavenging activity was calculated as follows:

$$DPPH\;scavenging\;rate(\%)=\left(1-\frac{A_{2}-A_{1}}{A_{0}}\right)\times 100$$ (2)

Where A_0_ is the absorbance of the control solution when the sample is replaced by distilled water, A_1_ is the absorbance of the sample solution when DPPH-ethanol is replaced by anhydrous aqueous alcohol, and A_2_ is the absorbance of the test sample solution.

### Hydroxyl Radical Scavenging Capacity

Hydroxyl radical scavenging capacity was estimated following a slightly modified method of Chen *et al*. [24]. 0.5 ml SDF sample solution of different concentrations was mixed with 0.5 ml FeSO4 (6.0 mM), 0.5 ml salicylic acid-ethanol solution (6.0 mM) and 0.5 ml H_2_O_2_ (6.0 mM). Then the mixture was incubated in a 37°C water bath for 30 min, and the absorbance was measured at 510 nm. The hydroxyl radical scavenging capacity was calculated as follows:

$$\cdot OH\;scavenging\;rate(\%)=\left(1-\frac{A_{2}-A_{1}}{A_{0}}\right)\times 100$$ (3)

Where A_2_ is the absorbance value of the sample solution, A_1_ is the absorbance value of the sample solution with distilled water instead of H_2_O_2_, and A_0_ is the absorbance value of the sample solution with water instead.

### Statistical Analysis

All experiments were repeated three times. Statistical analyses were performed with SPSS 17.0 statistical software, Origin 8.0, and Design Expert 13.0. Data from all samples were analyzed by analysis of variance (ANOVA) and Duncan's multiple tests. Results were recorded as mean ± standard deviation (SD). *p* < 0.05 was considered to be statistically significant.

## Results and Discussion

### Screening of Fermentation Strains

Fermentation modification relies on the enzyme system produced by the strain itself to degrade IDF and realize the conversion to SDF. Through the determination of the fermentation ability of 14 *A. niger* strains (Table 1), it could be demonstrated that *A. niger* F0607 had the highest cellulase activity (3.23 U/ml) and SDF yield (8.35%) compared to other strains tested, and the xylanase activity was maintained at a high level (102.96 U/ml). The selection of strains is not only determined by their ability to produce a variety of biologically active enzymes but also requires high cellulase activity, which has a significant degradation effect on IDF. Therefore, it was finally determined that *A. niger* F0607, with high cellulase activity and more significant degradation of IDF, was selected as the fermentation strain for the production of corn cob DF.

### Fermentation Condition Optimization of DF from Corn Cob

The fermentation conditions were firstly optimized using a one-way experiment with SDF yield as the evaluation index, and the results of data analysis were shown in Fig. S1. Excessive material-liquid ratio in the fermentation process will not only reduce the fermentation capacity of the bacteria but also increase the cost. Considering the factors of energy saving, environmental protection and economy in the actual production process as well as the operability of the laboratory, therefore, according to the results of the one-way test, the Box-Behnken experimental design principle was applied, and the inoculum concentration, time and temperature were selected as independent variables in a three-factor, three-level test. The experimental design and results were shown in Table 2. Data analysis obtained the regression equation between the three factors:

$$Y=11.62+0.3125X_{1}+0.5287X_{2}+0.1838X_{3}+0.035X_{1}X_{2}+0.13X_{1}X_{3}-0.625X_{2}X_{3}-0.5567X_{1}^{2}-0.7193X_{2}^{2}-0.6293X_{3}^{2}$$ (4)

Where Y-the yield of SDF (%); X_1_-Inoculum concentration (%); X_2_- Fermentation temperature (°C); X_3_-Fermentation time (d).

**Fig. 1:**
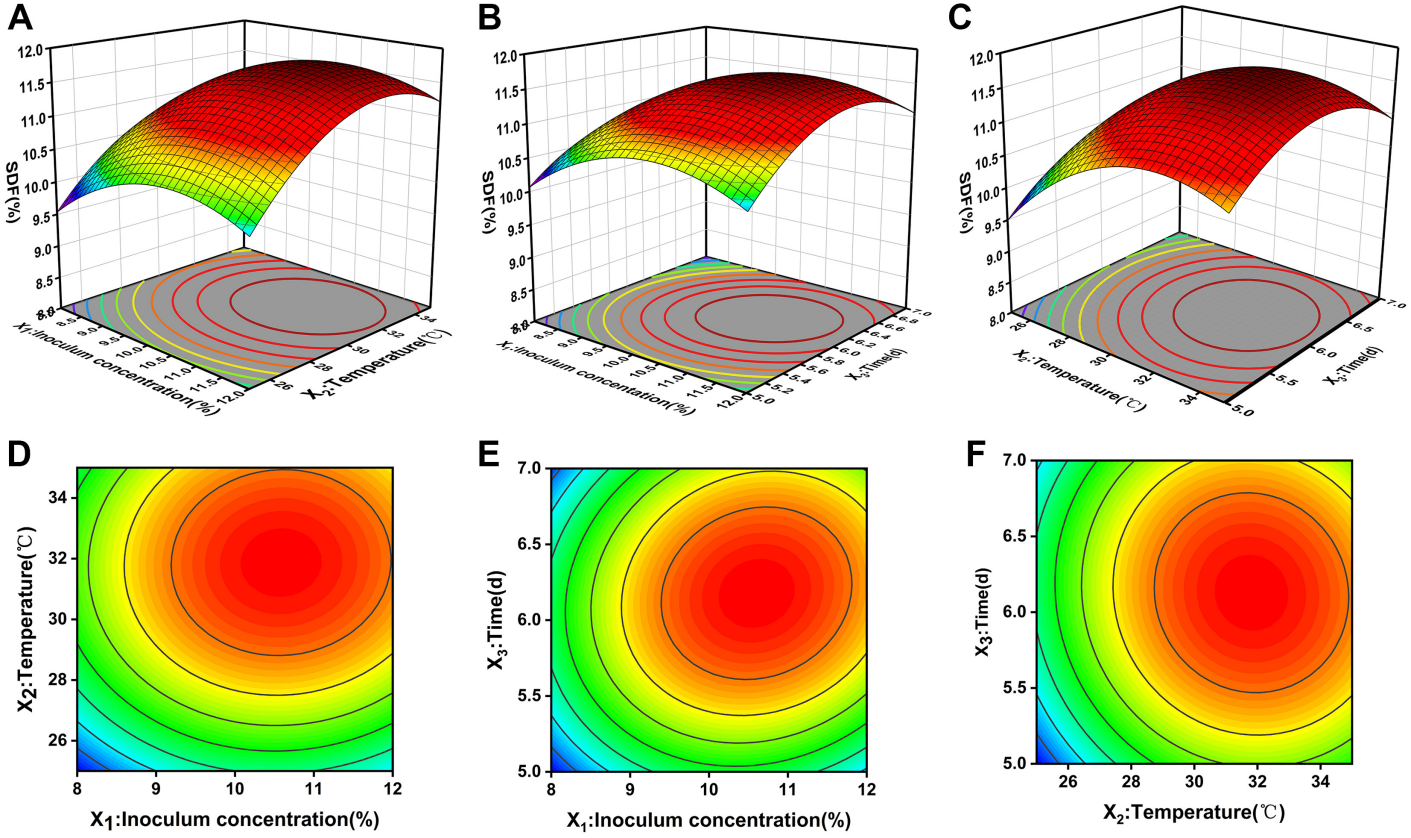
Response surface and contour plots showing the interactive effects of factors on SDF yield. (**A**) Response surface plot of the interaction between inoculation concentration and temperature. (**B**) Response surface plot of the interaction between inoculum concentration and time. (**C**) Response surface plot of the interaction between temperature and time. (**D**) Contour plots of the interaction between inoculation concentration and temperature. (**E**) Contour plots of the interaction between inoculum concentration and time. (**F**) Contour plots of the interaction between temperature and time.

The ANOVA results of the model were shown in Table S1. The quadratic equation model was highly significant at *p* < 0.01, indicating that the selected model was reliable. The 3D response surface of the model (Fig. 1) showed that the magnitude of the effect of the three factors on the yield of SDF from corn cobs was fermentation temperature (X_2_) > inoculum concentration (X_1_) > fermentation time (X_3_), in that order. Among them, the contour plot of two factors, X_1_ (inoculum concentration) and X_2_ (fermentation temperature), showed an elliptical shape with the most significant interaction, which is consistent with the results in the ANOVA table.

Box-Behnken response surface test predicted the highest SDF yield of 11.77% when the inoculum concentration was 10.60%, the fermentation temperature was 31.80°C, and the fermentation time was 6.17 d. The predicted results were verified by conducting three experiments on the optimal process parameters proposed by the numerical model. The actual experimental result was 11.91%, which was closer to the theoretical value, indicating that the experimental results were in good agreement with the model. Considering the actual operation, the optimal fermentation conditions were determined as 11% inoculum concentration, 32°C fermentation temperature, and 6 d.

As shown in Table 3, the DF content of corn cob is up to 82.62%, which can be considered as a good source of DF. The SDF content was 2.34% with a low activity value; however, the SDF of corn cob significantly increased to 11.92% by fermentation with *A. niger*. After fermentation, the content of IDF significantly decreased to 62.47%, and the SDF/TDF increased from 2.91% to 19.08%. The enzyme system produced by the strain can degrade IDF in corn cob and transform it into SDF through fermentation modification, thus improving the extraction of corn cob SDF. Therefore, *A. niger* fermentation can improve the quality of DF of corn cob.

### Fermentation Process Dynamic Monitoring

Fig. S2 demonstrates the dynamic fermentation process of SDF from corn cobs fermented by *A. niger*, and through monitoring the parameters during the fermentation process, we could then explore the changing pattern of high-yield SDF from corn cobs. The yield of SDF increased slowly in the early stage of fermentation, reaching a maximum of 0.11 g/g on the 6th day (Fig. S2A). However, it then decreased as the nutrients were depleted and possibly used by the strain. Meanwhile, the corn cob IDF content showed a decreasing trend with slight fluctuations later in fermentation.

The activities of cellulase and xylanase exhibited a pattern of rapid increase followed by a decline as the fermentation time progressed (Fig. S2B). The maximum enzyme activity was observed on the fourth day of fermentation, with cellulase activity reaching 2.80 U/ml and xylanase activity reaching 138.24 U/ml. This indicated that during the rapid adaptation of *A. niger* to mass growth and reproduction, some cellulose-degrading enzymes (including cellulases, xylanases, etc.) can be induced by using corn cob as the main component of the medium [25]. The amylase reached its peak of 1.44 U/ml on the fourth day of fermentation, exhibiting similar characteristics throughout the process.

Reducing sugar content increased rapidly during the initial stages of fermentation, peaking at 942.67 μg/ml on the third day. As fermentation progressed, reduced sugar content gradually decreased and showed slight fluctuations towards the later stages; however, these differences were not statistically significant (Fig. S2C). The total sugar content changed irregularly during the whole fermentation process, which may be attributed to the degradation of starch by amylase produced by the strain and the dynamic change of carbohydrate consumption by the strain itself. During the initial stage of fermentation (1-4 days), there was a gradual increase in soluble protein content, although this difference was not statistically significant. Starting from the 4th day, there was a steady increase in soluble protein content, reaching 2.49 mg/ml on the 7th day (Fig. S2D). This might be due to the production of proteases during fermentation, which enables the strain to synthesize proteins at a faster rate than metabolic consumption in the substrate [26]. This also indicated that the fermenting strain grew vigorously within 4-6 days and the secreted enzyme system was more capable of SDF production.

### Monosaccharide Composition and Structural Characterization of SDF

**Monosaccharide Composition of SDF**

The monosaccharide proportion in the SDF of corn cob prepared by *A. niger* had changed. Compared with B-SDF, F-SDF showed a new monosaccharide component (rhamnose) with 8.09%. Additionally, the mannose content increased from 3.77% to 25%, while other monosaccharides such as galacturonic, glucose, galactose, xylose and arabinose were significantly reduced or disappeared. The increase of rhamnose indicated that pectin degraded during fermentation, while the decrease of monosaccharides like galactose and glucose suggested that xylanase was secreted during *A. niger* fermentation played a crucial role in hemicellulose degradation [27].

### SEM

**Fig. 2:**
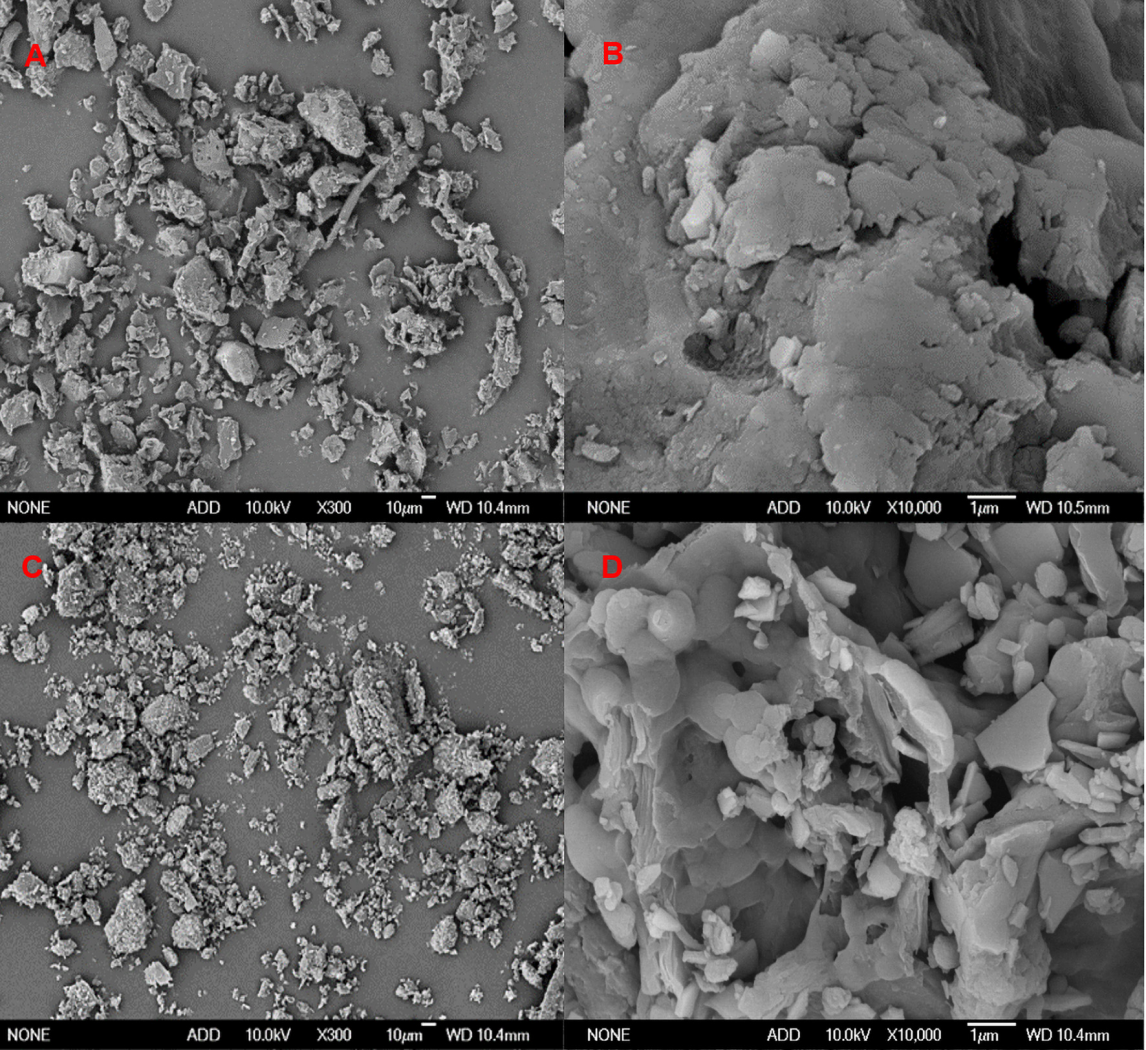
SEM micrographs of B-SDF and F-SDF. (**A**) and (**B**), the images of B-SDF were at magnifications of 300× and 10000×. (**C**) and (**D**), the images of F-SDF were at magnifications of 300× and 10000×. B-SDF: Unfermented corn cob soluble dietary fiber, F-SDF: Fermented corn cob soluble dietary fiber.

SEM is a crucial tool for investigating the microstructure of DF. In this study, the microstructure of SDF before and after the fermentation of corn cob was observed by SEM (Fig. 2). Comparing the two micrographs at the same magnification, it could be seen that B-SDF exhibited a larger overall particle size and an irregular blocky structure of varying sizes. The surface of B-SDF appeared smooth and dense (Fig. 2A and 2B). In contrast, F-SDF exhibited a lamellar structure accompanied by a distinct crumbly morphology. Its surface appeared to be sparse and porous (Fig. 2C and 2D). After fermentation, enzymatic action caused changes in the microstructure and molecular size of F-SDF. The original straight and branched chain structures in the corn cob fiber structure were hydrolyzed by enzymes such as cellulase and hemicellulase produced by the bacterium, resulting in glycosidic bonds cleavage of dietary fiber, a reduction in polymerization degree, and a decrease in molecular mass. Partial conversion of IDF to SDF in corn cobs was achieved [2]. Changes in the microstructure of DF will affect its functional properties and its application in food [28]. The loose structure is conducive to the adsorption properties of DF, such as WSC, WHC, OHC, and CAC [8]. It was hypothesized that the corn cob SDF prepared by *A. niger* fermentation had better physicochemical and functional properties.

### FT-IR

**Fig. 3:**
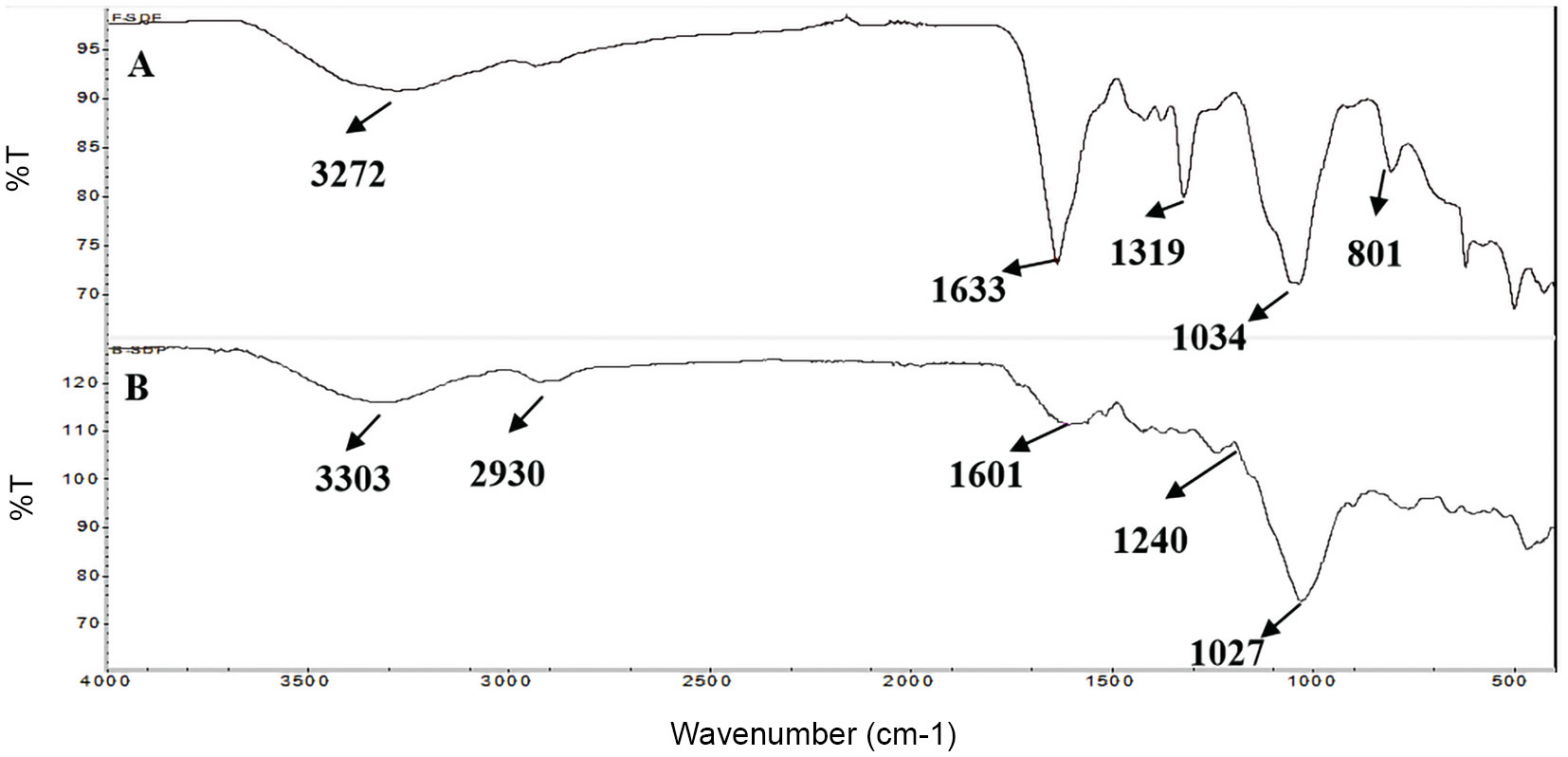
FTIR spectra of the B-SDF and F-SDF. (**A**) FTIR spectra of the B-SDF. (**B**) FTIR spectra of the F-SDF. B-SDF: Unfermented corn cob soluble dietary fiber, F-SDF: Fermented corn cob soluble dietary fiber.

The FT-IR spectra for B-SDF and F-SDF are shown in Fig. 3. The spectra of B-SDF detected absorption peaks at 3272, 1633, 1319, 1034, and 890 cm^-1^, corresponding to the -OH stretching vibration, C=O stretching vibration, C-H stretching vibration, and C-O stretching vibration and β-glycosidic bonds stretching vibration, respectively (Fig. 3A). The spectra of F-SDF detected absorption peaks at 3303, 2930, 1601, 1240, and 1027 cm^-1^ correspond to -OH stretching vibration, stretching vibration of C-H and -CH_2_-, C=O stretching vibration, C-H bending vibration, and C-O stretching vibration, respectively (Fig. 3B) [3, 26]. After fermentation, the band shifted from 3237 cm−1 to 3303 cm−1, probably due to the increase of free -OH produced from the hydrolysis of cellulose and hemicellulose due to the fermentation process. The weak peak of about 1601 cm−1 was characteristic bending or stretching of aromatic hydrocarbons of lignin [29].

The FT-IR spectra results indicated the presence of characteristic absorption peaks of polysaccharides in both B-SDF and F-SDF, with similar functional groups. The difference lied in the altered absorbance or wave number of certain characteristic absorption peaks in F-SDF. The results suggested that fermentation effectively degraded part of cellulose, hemicellulose, and lignin, producing numerous cellulose disaccharides and short-chain sugars. It also altered the aggregation structure of cellulose and formed more oil-philic groups, resulting in higher physiological and biochemical activities for F-SDF compared to B-SDF [28, 29].

### SDF Functional Properties

**WHC, WSC and OHC**

WSC is an important indicator of the physicochemical properties of DF. According to Table 3, The initial WS was obviously improved by fermentation. The WS of SDF before and after fermentation increased from 0.29 g/g to 0.53 g/g by 85.96%. The porous structure of SDF allows it to absorb water and cause swelling, which improved the WSC [28]. On the other hand, enzymes generated during fermentation disrupted the connections between cellulose and hemicelluloses, thereby exposing more hydrogen bonds and dipole forms that contributed to an improvement in solubility [30].

High WHC of DF could prevent food shrinkage and change the viscosity of food. WHC was the embodiment of good functional properties of DF. As shown in Table 3, WHC increased from 3.49 g/g to 4.73 g/g by fermentation treatment. Some studies have found that DF with high WHC can be attributed to the hydrophilic groups of polysaccharides and are also closely related to factors such as content, particle size, surface properties, and the source of SDF [19]

The OHC of DF is important in food applications, for example, DF with high OHC can stabilize high-fat foods and dairy products. The OHC of F-SDF was higher than that of B-SDF, which was 5.10 g/g and 6.97 g/g for B-SDF and F-SDF, respectively. The increase in the oil-holding power of F-SDF might be due to its loose and porous structure. It might also be related to the presence of arabinoxylans, pectins, and arabinogalactans in SDF, which may contribute to the adsorption and removal of saturated and unsaturated lipids from SDF due to their strong affinity for lipid substances [31]. High OHC interferes with the intestinal absorption of dietary lipids, thus helping to control body weight and abnormal lipid profiles.

### GAC

**Fig. 4:**
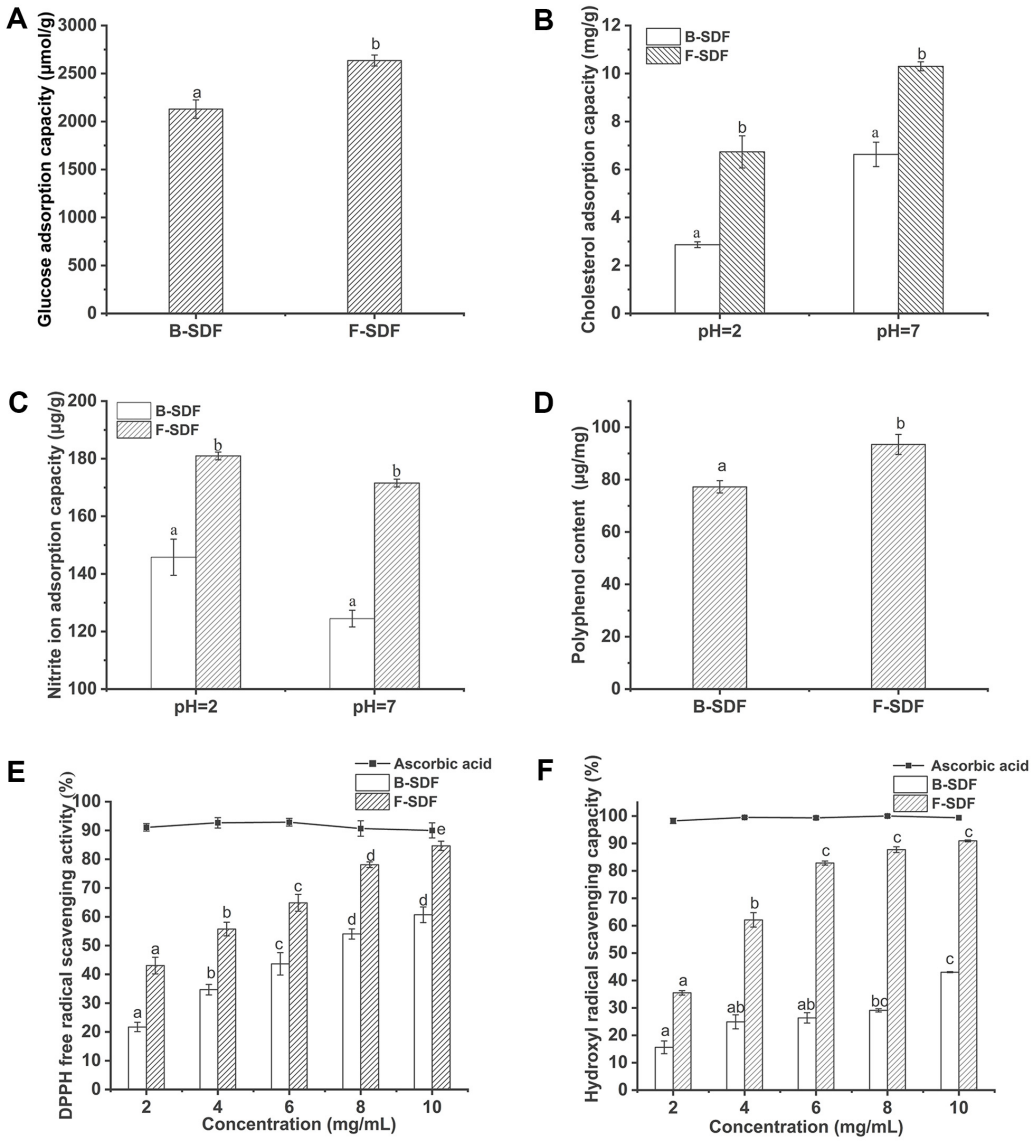
Functional properties of SDF. (**A**) Glucose adsorption capacity. (**B**) Cholesterol adsorption capacity. (**C**) Nitrite adsorption capacity. (**D**) Polyphenol content. (**E**) DPPH free radical scavenging activity. (**F**) hydroxyl radical scavenging activity. ^a-c^Values marked with different letters differ significantly (*p* < 0.05). B-SDF: Unfermented corn cob soluble dietary fiber, F-SDF: Fermented corn cob soluble dietary fiber.

The ability of DF to absorb glucose can delay or reduce the digestion and absorption of glucose in the gastrointestinal tract, which plays an important role in controlling blood sugar. The SDF fermented by *A. niger* could significantly improve the GAC, and the GAC of F-SDF was 2635.57 μmol/g, which was 23.78% higher than that of B-SDF (2129.18 μmol/g) (Fig. 4A). It has been suggested that the enhanced adsorption capacity of glucose may be related to viscosity, porosity and special surface structure [20]. Combined with the structural analysis, the increased GAC of F-SDF may be due to the degradation of fiber surface by cellulase and xylanase produced by fermentation, which loosens the fiber structure and increases the pore size, making it easier for glucose molecules to be absorbed into the fiber. In contrast, the unfermented DF have a dense structure and most of the key functional groups are surrounded by the internal structure of the fibers, resulting in their inability to play an effective role [29].

### CAC

pH is an important factor affecting the cholesterol adsorption capacity of DF, therefore, pH 2.0 (simulated gastric environment) and pH 7.0 (simulated small intestine environment) were chosen to investigate the CAC of B-SDF and F-SDF, respectively, and the results are shown in Fig. 4B. The CAC of DF rose as the pH, which suggested that the CAC of F-SDF was stronger in the simulated small intestinal environment than in the simulated gastric environment The CAC of F-SDF was increased by 55.30% compared with B-SDF at pH 2.0, which was 2.87 mg/g and 6.74 mg/g for B-SDF and F-SDF, respectively. This might be due to the loose structure and the increase of the specific surface area of F-SDF, which lead to the easier penetration of fat-soluble substances into DF [21].

### NAC

Fig. 4C shows the adsorption of nitrite ions by B-SDF and F-SDF at pH 2.0 (simulated stomach environment) and pH 7.0 (simulated small intestine environment). At pH 2.0, the NAC of F-SDF reached 180.96 μg/g, which was 24.12% higher than that of B-SDF, which was 145.79 μg/g. At pH 7.0, the NAC of B-SDF and F-SDF was 124.48 μg/g and 171.54 μg/g, respectively. The pH had a significant effect on the nitrite adsorption capacity of SDF. The NAC of SDF was higher at pH 2.0 than at pH 7.0 in all cases, indicating that the adsorption capacity of SDF in the stomach was higher than that in the intestine. Nitrite can react to form N-nitroso compounds, many of which have been proved to be carcinogenic in animals [3]. SDF has a blocking effect on the synthesis of carcinogens and should have a preventive effect on the development of cancer. Therefore, SDF can be used as a nitrite scavenger. The property of high nitrite adsorption capacity provides a possibility for F-SDF to be used as a functional food to prevent the occurrence of gastric cancer [1].

### Antioxidant Properties

As shown in Fig. 4D, the total phenolic content (TPC) of F-SDF was 93.45 mg/g, which increased by 20.96%compared with B-SDF. This suggests that fermentation may facilitate the release of phenols [22]. With ascorbic acid as the positive control, the scavenging ability of B-SDF and F-SDF on DPPH free radical and hydroxyl free radical were less than that of ascorbic acid in the experimental concentration range (2-10 mg/ml), but both scavenging ability of DPPH free radical and hydroxyl free radical increased with the increase of SDF concentration (Fig. 4E and 4F). In addition, the scavenging ability of F-SDF on DPPH free radical and hydroxyl free radical were significantly higher than that of B-SDF. The DPPH radical scavenging capacity of F-SDF reached 84.03% at the SDF concentration of 10.0 mg/ml, which was 38.47% higher than that of B-SDF. At SDF 6.0 mg/ml, the hydroxyl radical scavenging rate of F-SDF was 2.14 times higher than that of B-SDF, and at SDF 10.0 mg/ml, the scavenging rate of hydroxyl radicals by F-SDF reached 90.93%, which was close to that of ascorbic acid.

The above results indicated that the reducing ability of corn cob SDF was enhanced by *A. niger* fermentation. This may be related to the higher content of polyphenols in F-SDF. It has been shown that there is a positive correlation between polyphenol content and DPPH free radical scavenging activity [23]. Fermentation may contribute to the release and dissolution of phenolic substances, and the increased content of polyphenols promotes the free radical scavenging ability of SDF [11].

## Conclusion

The results showed that corn cob DF with excellent food safety can be produced by fermentation of *A. niger*. The structure, physicochemical properties, and functional characteristics of DF were also significantly improved. The corn cob SDF could not only be used as a functional component in food but also realize the conversion of low-value-added products corn cob to high-value-added DF. In our study, the yield of SDF was 11.92% and the SDF/TDF was 19.08% by fermentation, making corn cobs a high-quality DF source. The fermentation of *A. niger* caused the DF to form a loose and porous structure, which in turn significantly enhanced the physicochemical properties of corn cob SDF, such as good WS, WHC, OHC, GAC, CAC, and NAC. Fermentation contributes to the release of phenolics, thus enhancing the reducing ability of corn cob SDF, including DPPH radical scavenging capacity and hydroxyl radical scavenging capacity, and obtaining higher physiological activity. The fermentation of corn cobs by *A. niger* as an edible fungus improves the structural characteristics and functional properties of DF, enabling the preparation of highly active corn cob DF products that can be applied in food products.

## Supplemental Materials

Supplementary data for this paper are available on-line only at http://jmb.or.kr.



## Figures and Tables

**Table 1:** Determination results of enzyme activity and SDF.

Strain	Cellulase activity (U/ml)	Xylanase activity (U/ml)	SDF (%)
F0607	3.23	102.96	8.35
F0609	3.11	98.66	7.29
F0612	1.60	128.45	5.43
3.1858	2.38	74.50	4.93
3.2103	2.29	64.21	2.69
3.2113	2.55	88.34	4.75
3.213	1.58	72.23	6.07
3.2169	2.59	74.78	3.60
3.875	2.52	59.84	2.88
3.489	2.69	92.91	3.38
3.4309	2.57	66.33	7.56
3.2133	2.45	108.62	7.38
3.3928	2.32	86.05	7.94
3.876	2.80	78.51	6.89

**Table 2:** Experimental scheme and results of Box-Behnken design.

Runs	Factors			SDF (/%)
	X_1_	X_2_	X_3_	
1	8	25	6	9.68
2	12	25	6	9.95
3	8	35	6	10.66
4	12	35	6	11.07
5	8	30	5	9.82
6	12	30	5	10.47
7	8	30	7	10.13
8	12	30	7	11.30
9	10	25	5	9.59
10	10	35	5	10.78
11	10	25	7	9.88
12	10	35	7	10.82
13	10	30	6	11.85
14	10	30	6	11.40
15	10	30	6	11.58
16	10	30	6	11.48
17	10	30	6	11.77

Note: X_1_: Inoculum concentration (%), X_2_: Fermentation temperature (°C), X_3_: Fermentation time.

**Table 3:** The composition and physicochemical properties of dietary fiber.

Samples	SDF (%)	IDF (%)	TDF (%)	SDF/IDF (%)	WSC (g/g)	WHC (g/g)	OHC (g/g)
B-SDF	2.34 ± 0.085^a^	80.28 ± 0.056^a^	82.62 ± 0.071^a^	2.91 ± 0.073^a^	0.285 ± 0.065^a^	3.485 ± 0.165^a^	5.100 ± 0.18^a^
F-SDF	11.92 ± 0.034^b^	62.47 ± 0.067^b^	74.39 ± 0.051^b^	19.08 ± 0.047^b^	0.530 ± 0.010^b^	4.725 ± 0.095^b^	6.965 ± 0.325^b^

B-SDF: Unfermented corn cob soluble dietary fiber, F-SDF: Fermented corn cob soluble dietary fiber. Each value was expressed as the mean ± SD (*n* = 3). The results were significant (*p* < 0.05).
