# Supplementary figures and images for: Preparation and Improvement of Physicochemical and Functional Properties of Dietary Fiber from Corn Cob Fermented by Aspergillus niger
Source: J Microbiol Biotechnol. 2023 Oct 16;34(2):330–9. doi: 10.4014/jmb.2308.08010 (PMC10940746; doi:10.4014/jmb.2308.08010)

## Graphical Abstract

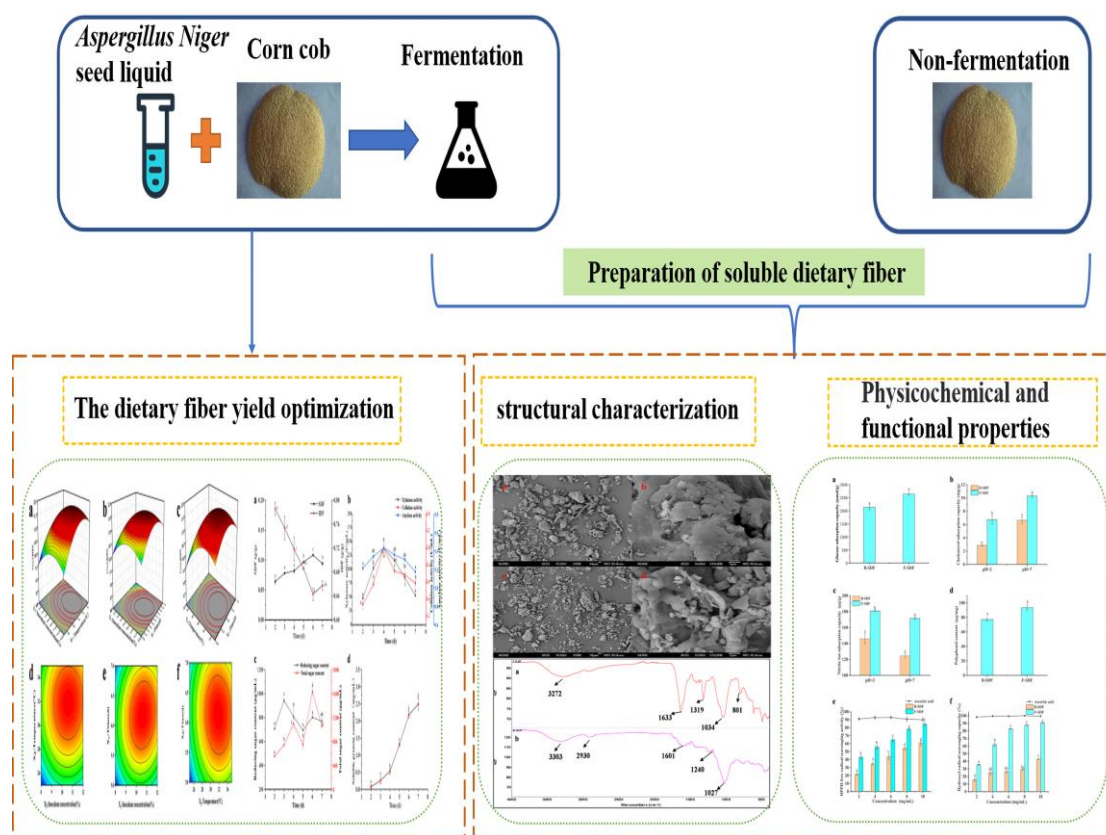

Supplement: Supplementary file 1 [file jmb-34-2-330-supple.pdf]
